# Temporal Changes of Virus-Like Particle Abundance and Metagenomic Comparison of Viral Communities in Cropland and Prairie Soils

**DOI:** 10.1128/mSphere.01160-20

**Published:** 2021-06-02

**Authors:** Carolyn R. Cornell, Ya Zhang, Joy D. Van Nostrand, Pradeep Wagle, Xiangming Xiao, Jizhong Zhou

**Affiliations:** a University of Oklahoma, Department of Microbiology and Plant Biology, Norman, Oklahoma, USA; b University of Oklahoma, Institute for Environmental Genomics, Stephenson Research and Technology Center, Norman, Oklahoma, USA; c University of Oklahoma, School of Civil Engineering and Environmental Sciences, Norman, Oklahoma, USA; d USDA, Agricultural Research Service, Grazinglands Research Laboratory, El Reno, Oklahoma, USA; e Lawrence Berkeley National Laboratory, Earth and Environmental Sciences, Berkeley, California, USA; f School of Environment, Tsinghua University, State Key Joint Laboratory of Environment Simulation and Pollution Control, Beijing, China; University of Illinois at Urbana-Champaign

**Keywords:** agriculture, microbial ecology, soil virus, viral ecology, viral metagenomics

## Abstract

During the last several decades, viruses have been increasingly recognized for their abundance, ubiquity, and important roles in different ecosystems. Despite known contributions to aquatic systems, few studies examine viral abundance and community structure over time in terrestrial ecosystems. The effects of land conversion and land management on soil microbes have been previously investigated, but their effects on virus population are not well studied. This study examined annual dynamics of viral abundance in soils from a native tallgrass prairie and two croplands, conventional till winter wheat and no-till canola, in Oklahoma. Virus-like particle (VLP) abundance varied across sites, and showed clear seasonal shifts. VLP abundance significantly correlated with environmental variables that were generally reflective of land use, including air temperature, soil nitrogen, and plant canopy coverage. Structural equation modeling supported the effects of land use on soil communities by emphasizing interactions between management, environmental factors, and viral and bacterial abundance. Between the viral metagenomes from the prairie and tilled wheat field, 1,231 unique viral operational taxonomic units (vOTUs) were identified, and only five were shared that were rare in the contrasting field. Only 13% of the vOTUs had similarity to previously identified viruses in the RefSeq database, with only 7% having known taxonomic classification. Together, our findings indicated land use and tillage practices influence virus abundance and community structure. Analyses of viromes over time and space are vital to viral ecology in providing insight on viral communities and key information on interactions between viruses, their microbial hosts, and the environment.

**IMPORTANCE** Conversion of land alters the physiochemical and biological environments by not only changing the aboveground community, but also modifying the soil environment for viruses and microbes. Soil microbial communities are critical to nutrient cycling, carbon mineralization, and soil quality; and viruses are known for influencing microbial abundance, community structure, and evolution. Therefore, viruses are considered an important part of soil functions in terrestrial ecosystems. In aquatic environments, virus abundance generally exceeds bacterial counts by an order of magnitude, and they are thought to be one of the greatest genetic reservoirs on the planet. However, data are extremely limited on viruses in soils, and even less is known about their responses to the disturbances associated with land use and management. The study provides important insights into the temporal dynamics of viral abundance and the structure of viral communities in response to the common practice of turning native habitats into arable soils.

## INTRODUCTION

Viruses have been making their way to the forefront of ecological research for their significant roles in marine and terrestrial ecosystems, being found everywhere that life exists. Most knowledge on viral ecology has been generated from the study of natural virus populations in marine and freshwater ecosystems, where viruses have been shown to mediate horizontal gene transfer (1), help drive biogeochemical nutrient cycling (2), and play a central role in controlling the total abundance, population dynamics, and evolution of their hosts (3, 4). It has been estimated that viruses may be the most abundant biological entity on the Earth at 10^31^ viruses (5), with soils providing one of the greatest reservoirs (6). As a result, it is now predicted that viruses have equal ecologically valuable roles in terrestrial environments. Soils provide a more diverse habitat for viruses than aquatic environments due to their wide compositional range, spatial heterogenicity in terms of physicochemical properties, and management practices, allowing viruses to be exposed to many unique ecological pressures that are not present in aquatic systems (7–10). Understanding the response of virus communities to such pressures is critical to the knowledge of soil ecology and important for ecosystem sustainability.

Natural land conversion is a prevalent practice that results in distinct effects on the soil characteristics and function of terrestrial ecosystems. Specifically, agricultural cultivation has significantly changed land use across North America, resulting in the depletion of native tallgrass prairies to 4% of their original land coverage (11, 12). The majority of new croplands in the United States were initially grasslands with roughly a fourth of the converted land planted with wheat (13), which is now the dominant annually cropped plant in the Southern Plains. Grasslands are important for preventing erosion, acting as carbon sinks and as a source of nitrogen fixation (14). Converting previously natural land into arable soils results in above and below ground species loss, allowing species invasion, as well as introducing disturbances to soil and biological processes (15–17). Together, these anthropogenic activities act as environmental stressors greatly impacting soil ecosystems with little known about the effects on virus populations. Since viruses are highly abundant and influence microbial hosts, it is important to understand the impacts of land use and management practices on the soil viral community.

Estimates of viruses in terrestrial environments are the first step to identifying virus significance in soils since organisms that are present in large numbers generally play important roles in ecosystem function. Transmission electron microscopy (TEM) and epifluorescence microscopy (EFM) have been used in aquatic systems to show a range in viral abundance of 10^4^ to 10^8^ ml^−1^, providing evidence that viruses are a prevalent component of marine and freshwater environments (18, 19). Advance in epifluorescence microscopy resulted in an approach to directly visualize virus particles in marine systems (20, 21). These previous discoveries have resulted in the development of methods to mechanically extract, microscopically enumerate, and quantify viruses from soils (22–24). Virus-like particle (VLP) abundance ranging from 10^7^ to 10^9^ VLPs g^−1^ soil has been observed in a diverse range of sites and soil types (3, 23–25). For example, more nutrient-rich soils found in forests and pastures generally have a higher viral abundance than soils from croplands and extreme locations such as Antarctica (3, 24, 25). The VLP abundance often exceeds bacterial abundance, with it being thought that viral abundance is dependent on the productivity of the hosts, as well as viral persistence (3, 24, 26), but few studies examine these dynamics at seasonal or annual timescales in soils. While studies in marine environments have presented clear temporal dynamics in viral abundance and community structure (27, 28), limited research leaves much to be discovered about the spatiotemporal changes of viruses in soils of terrestrial ecosystems.

To compare differences of viral populations, it is fundamental to have an accurate assessment community composition. Investigations have come to rely on high-throughput sequencing to evaluate diversity, population structure, and potential functional importance of whole viral assemblages. As studies of marine systems have reported a diverse population of DNA and RNA viruses (29–31), most terrestrial studies focus on dsDNA viruses or examine extreme landscapes such as polar (32) and desert regions (7, 33). Comparisons of viral communities between soil and aquatic environments have implied that distinct habitat types consist of distinct viral communities (30, 32, 34). With advances toward optimized methods for studying terrestrial viruses, recent studies in a thawing permafrost gradient recovered roughly 2,000 viruses approximately doubling the number of known genera in the RefSeq database at the time (35, 36) with the number of uncultivated virus genomes greatly surpassing the number of sequenced virus isolates in publicly available databases (37). Such studies demonstrate that metagenomic analysis of a single environmental gradient has the ability to greatly expand the knowledge of terrestrial viruses. It also emphasizes the importance of including viral abundance and viral community structure in studies to fully understand the dynamics of soil ecosystems in response to environmental changes.

The objective of this study was to determine whether there were temporal changes in virus and their potential bacterial host abundance in three differently managed Oklahoma soils. Experimental sites included a native tallgrass prairie (never tilled or cultivated), conventional till (CT) winter wheat, and no-till (NT) canola. By using data from multiple sites, we also aimed to determine whether the abundance of the viral communities was affected by increasing amounts of land management by examining the influence of soil and environmental factors on VLP abundance over the 1-year sampling period. Furthermore, metagenomic analysis was used to examine the impact of land use on viral community structure in soils of the native prairie and CT cropland, which aimed to exam whether viral abundance or community composition played a larger role in the observed changes in viral populations. Our results indicated that soil properties, plant canopy cover, and environmental factors such as air temperature, most of which are further controlled by land use and land management practices, are important in shaping virus-host interactions, along with virus and host abundance.

## RESULTS

### Soil, plant, and environmental properties.

**FIG 1:**
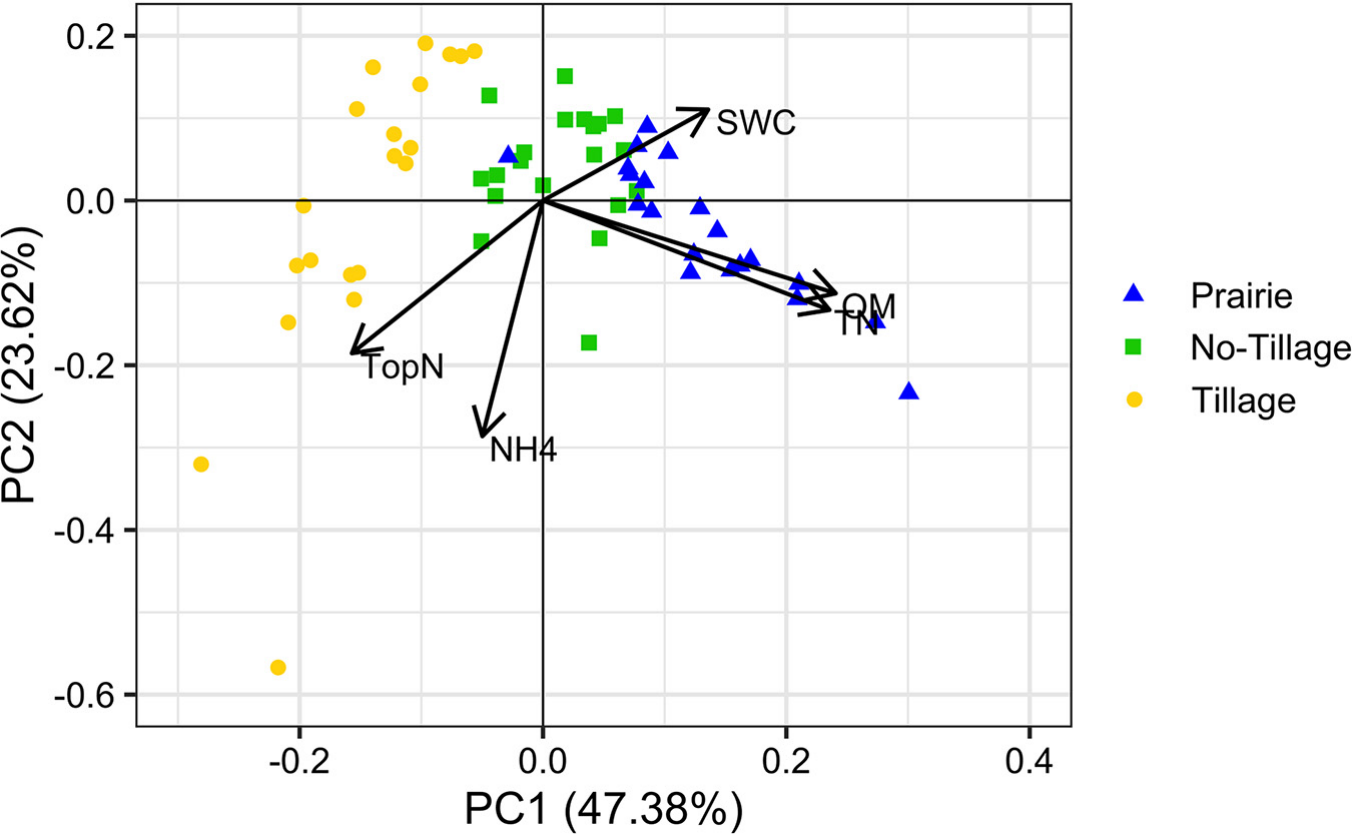
Comparison of soil properties that significantly varied between land use and land management based on principal component analysis. Study sites include native tallgrass prairie, no-till canola, and conventional till wheat. Soil properties data were collected from August 2016 to July 2017.

Land use and land management had considerable impact on soil properties (Fig. 1). All measured soil properties were significantly different between at least one set of sites (*P* < 0.05). Significant differences between field comparisons varied with specific soil properties. Organic matter (OM) and total nitrogen (TN) were significantly different (*P* < 0.001) in pairwise comparisons between all fields. OM and TN levels in soil decreased with increasing levels of management input. Croplands had significantly higher (*P* < 0.05) topsoil nitrate (TopN) compared to the native tallgrass prairie. The CT wheat field had TopN of 49 kg ha^−1^ on an average and was as high as 160 kg ha^−1^. The NT canola field had higher level of TopN (37 kg ha^−1^) compared to the native tallgrass prairie (13.5 kg ha^−1^) on average. Ammonium (NH_4_) levels were only marginally significantly lower (*P* = 0.059) in the NT cropland (13.9 kg ha^−1^), while NH_4_ levels were only slightly greater in the CT cropland at 24.0 kg ha^−1^ than in prairie soil at 22.4 kg ha^−1^, on average. Nitrogen fertilizer was applied in both croplands during planting, while native prairie was not fertilized.

From August 2016 to September 2017, monthly rainfall ranged from lows of 14.99 mm during November 2016 and highs of 227.08 and 252.22 mm during April 2017 and August 2017, respectively. Over the growing season of wheat and canola from October 2016 to May 2017, no severe drought was observed, with the sites receiving ∼508 mm of rain. Overall, CT wheat had the lowest soil water content (*P* < 0.001) of all the fields. The tallgrass prairie had soil water content (SWC) of 18% and the NT cropland SWC was 17% on average, whereas the CT cropland site had an average of 10% with SWC as low as 3%. However, lower SWC was recorded during winter (dormant period for the crops).

Air temperature reached a maximum during the summer months in August 2016 and July 2017 with minimum air temperatures during winter in December and January. Annual dynamics of soil temperature varied with sites and growing seasons, often differing based on land use type due to contrasting seasonality of crops and native prairie. Wheat was planted on September 12 and grazed from 15 November 2016 to 9 May 2017. Canola was planted on 3 October 2016 and harvested in June 2017. Plant biometrics measurements were taken during the fall 2016 and spring 2017 at both croplands. Higher values of leaf area index (LAI), biomass, and canopy cover percentage were observed before and after winter since both crops were dormant during winter. By mid-November, LAI reached ∼5 m^2^ m^−2^ for canola and ∼3 m^2^ m^−2^ for wheat, while canopy cover percentage was >95 for canola and >80 for wheat. Vegetation growth in croplands increased again with increasing air temperature in spring, with canopy cover percentage >70 in both fields and LAI of ∼3 and 3.5 m^2^ m^−2^ for canola and wheat, respectively, in April. Native prairie vegetation greened up in April and entered into senescence phase at the end of October. Croplands had higher soil temperatures during the summer compared to the tallgrass prairie because croplands were left fallow from June to September, while summer was peak growing season for the prairie.

### Temporal dynamics of VLP abundance and its influencing factors.

**FIG 2:**
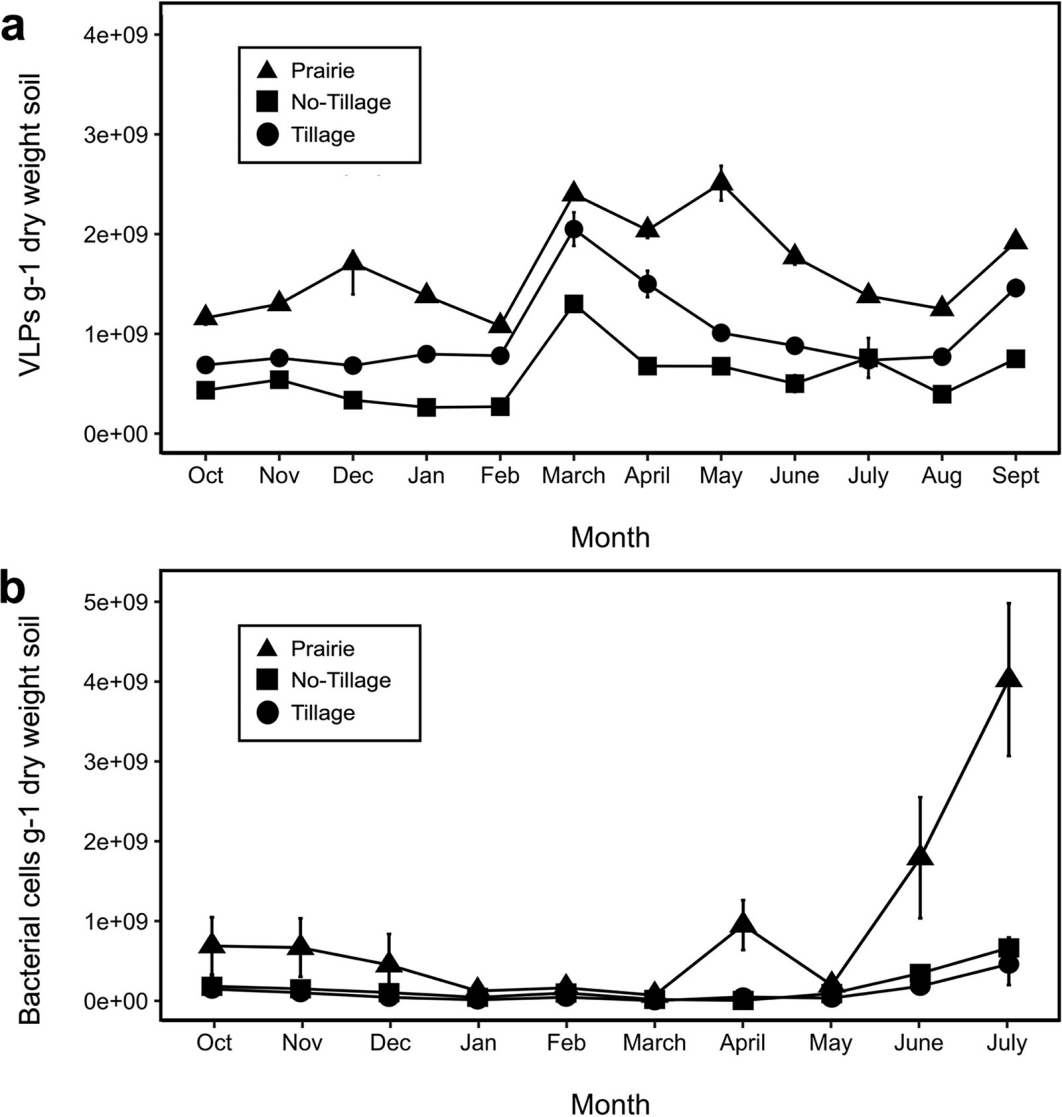
VLP and bacterial abundance between different land usage and land management. (a) VLP abundance over a 1-year sampling period from October 2016 to September 2017. VLP abundance was calculated based on the dry weight of soil. (b) Bacterial cell abundance at corresponding sampling dates for VLP samples. Only time points of bacterial abundance that overlap with VLP abundance are shown in the figure.

The VLP abundance was substantially altered due to land use and land management practices (*P* < 0.0001). Over the sampling period, VLP abundance ranged from 2.63 × 10^8^ to 2.51 × 10^9^ VLP g^−1 ^dry weight among the three sites (Fig. 2a). The greatest difference in abundance was observed between native prairie and CT wheat (*P* < 0.001). There was also a significant difference for VLP abundance between native prairie and NT canola (*P* = 0.001) and both croplands (*P* < 0.05). The average abundance was 1.66 × 10^9^ VLP g^−1^ in prairie soil, 1.01 × 10^9^ VLP g^−1^ in NT canola, and 5.75 × 10^8^ VLP g^−1^ in CT wheat. The tallgrass prairie had the greatest VLP abundance during all sampling months. The CT wheat had the lowest abundance except for July (fallow period) where VLP abundance was greater than that of the NT canola. Seasonal variations were observed with significant changes in abundance related to sampling month (*P* < 0.01) at all sampling sites. The shifts detected in the croplands overall followed the same seasonal dynamics with lower abundance observed during winter (December through February), and peak VLP abundance in March (i.e., the period of rapid vegetation growth with rise in temperature). This was supported by the most pairwise significant differences (*P* < 0.05) being observed for January and February in the winter and March and April in the spring. Prairie soil also had lower VLP abundance in February and elevated VLP abundance during the spring months, March through May (i.e., greening up and rapid growth of prairie vegetation) that was further supported significant pairwise difference (*P* < 0.05) in abundance between sampling months. The highest standard deviation was observed in the tallgrass prairie site at 4.80 × 10^8^ VLP g^−1^. In comparison, croplands had lower standard deviations of 4.31 × 10^8^ VLP g^−1^ and 2.88 × 10^8^ VLP g^−1^ at NT canola and CT wheat, respectively.

Spearman correlations were calculated to determine which soil, plant, and environmental factors potentially influenced VLP abundance for individual sites (Table 1). No highly significant correlations were observed between tallgrass prairie parameters and VLP abundance. SWC and VLP abundance at the prairie site had the strongest correlation, but it was not significant (rho 0.40, *P* = 0.0993). The NT canola had a significant negative correlation between VLP abundance and TopN (rho −0.65, *P* = 0.0204), and highly significant positive relationship between VLP abundance and leaf area index (rho 1.00, *P* < 0.001). The VLP abundance had correlations with several different factors at the CT wheat field. The VLP abundance showed a moderately strong correlation with air temperature (rho 0.49, *P* = 0.0531), and significant positive correlations with plant biometrics such as plant biomass, leaf area index, and canopy height. The only significantly negative correlation (rho −0.56) was overserved between ammonium and VLP abundance at the CT wheat field.

**TABLE 1:** Influence of soil, plant, and environmental factors on VLP abundance within fields based on Spearman correlations^*a*^

Parameter	Native prairie		No-till		Conventional till	
	Rho	*P*	Rho	*P*	Rho	*P*
Topsoil nitrate	0.01	0.5101	**−0.65**	**0.0204**	−0.37	0.1492
Organic matter	0.14	0.6504	−0.03	0.4669	0.15	0.3438
Total N	0.05	0.5539	−0.04	0.4527	−0.04	0.4561
NH_4_	0.07	0.4206	−0.12	0.6243	**−0.56**	**0.0449**
SWC	*0.40*	*0.0992*	0.04	0.4485	0.28	0.2215
Avg rain	0.22	0.2596	−0.09	0.3952	−0.09	0.3952
Min temp	0.09	0.3892	0.06	0.4314	*0.43*	*0.0834*
Avg temp	0.10	0.3767	0.03	0.4656	*0.49*	*0.0531*
Max temp	0.02	0.4785	−0.01	0.5086	*0.48*	*0.0591*
Avg soil temp	0.12	0.3685	−0.18	0.3508	0.47	0.1027
Plant biomass	–	–	0.40	0.3000	**1.00**	**<0.001**
LAI	–	–	**1.00**	**<0.001**	**1.00**	**<0.001**
Canopy cover	–	–	−0.50	0.3333	0.50	0.3333
Canopy ht	–	–	–	–	**1.00**	**<0.001**

a Correlation coefficients with *P* < 0.05 are indicated in boldface; coefficients with 0.1 > *P* > 0.05 are indicated in italics. Dashes (–) represent missing data. Units and abbreviations: topsoil nitrate (lkg/ha), organic matter (%), total nitrogen (%), NH_4_ (lkg/ha), gravimetric soil water content (%), daily average soil temperature at 6-cm depth, leaf area index (LAI), canopy height (cm), canopy cover (%), and dry plant biomass (kg/m^2^).

**FIG 3:**
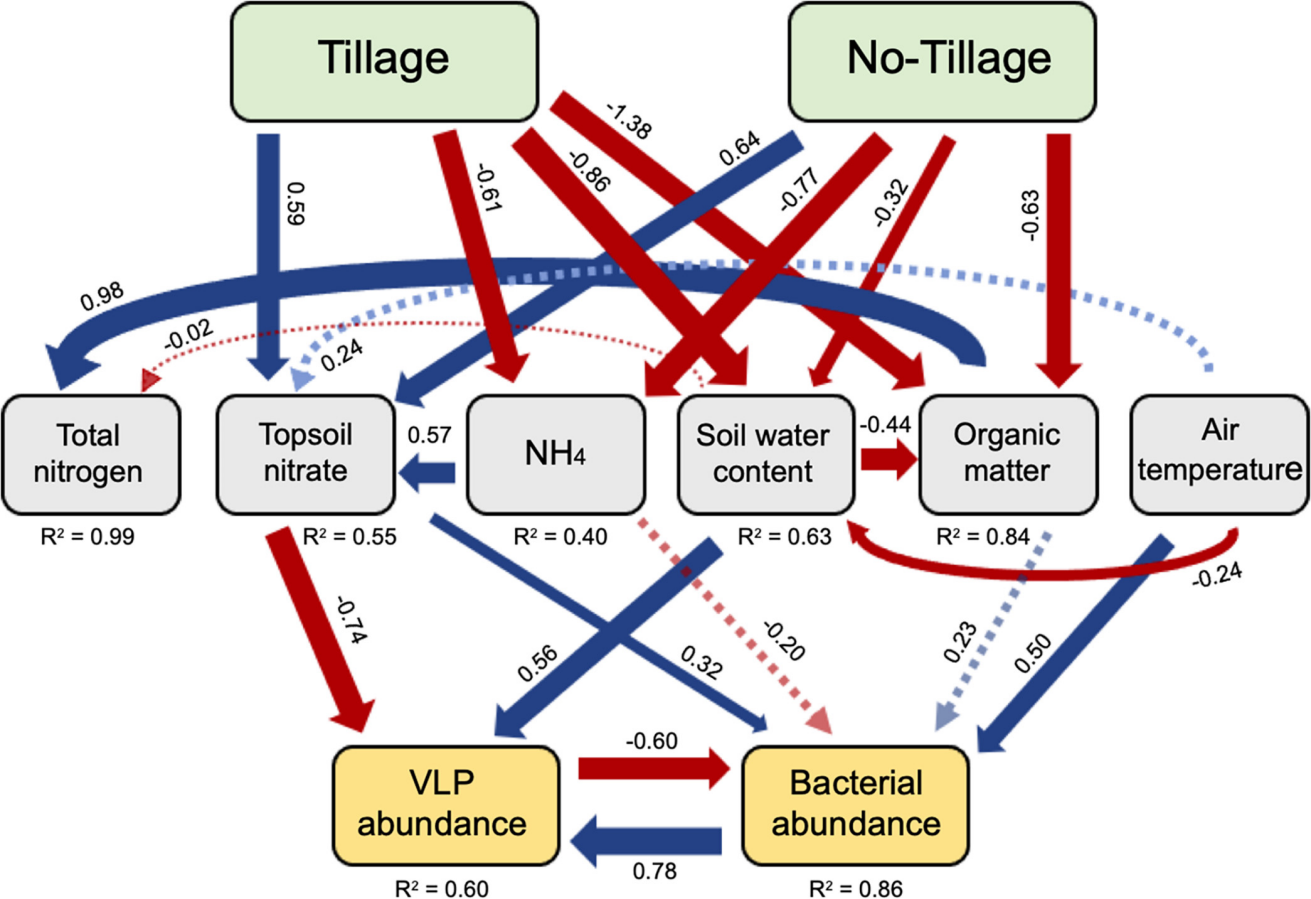
Relationship between VLP abundance, bacterial abundance, land management, and soil and environmental factors based on structural equation modeling. Solid arrows indicate factors that had *P* values of <0.05. Dashed arrows indicate factors with marginal nonsignificant relationships (*P* < 0.1). Red arrows represent negative relationships, while blue arrows represent positive relationships. Native tallgrass prairie was used as the control, with the two management practices acting as treatments.

Structural equation modeling (SEM) was used to further estimate the direct and indirect relationships between the soil variables and VLP abundance. SEM results were similar to those observed in the Spearman correlations (Fig. 3). The VLP abundance was indirectly influenced by land management practices that directly influenced TopN, and SWC. Bacterial abundance also had significant positive influence on the overall VLP abundance (*P* = 0.034). TopN was positively influenced by NH_4,_ air temperature, and land use, while SWC had a negative effect on nitrate levels. Lastly, SWC had significant negative relationships with land management and average air temperature with tillage land use having the strongest effect.

### Temporal dynamics of bacterial abundance and its influencing factors.

Bacterial abundance was significantly different (*P ≤ *0.001) among the sites ranging from 10^5^ to 10^9^ bacterial cells g^−1 ^dry weight (Fig. 2b). Bacterial abundance was significantly less in CT wheat (*P ≤ *0.001) and NT canola (*P ≤ *0.05) than that of the tallgrass prairie. On average, the tallgrass prairie had an abundance of 6.87 × 10^8^ cells g^−1 ^dry weight, followed by NT canola (1.37 × 10^8^ cells g^−1 ^dry weight) and CT wheat (7.50 × 10^7^ cells g^−1 ^dry weight). Both croplands had lower standard deviations (i.e., 1.88 × 10^8^ cells g^−1 ^dry weight for NT canola and 1.41 × 10^8^ cells g^−1 ^dry weight for CT wheat) than that of the tallgrass prairie (1.02 × 10^9^ cells g^−1 ^dry weight). Significant seasonal shifts (*P* < 0.05) were detected at all sampling sites. In the tallgrass prairie, bacterial abundance in spring was significantly different from that of summer and winter, while June and July were the most significantly differently from other sampling months. Spring was also significantly different than summer and winter in the NT canola site along with significant differences between bacterial abundance during summer and fall. Significant seasonal differences only occurred in bacterial abundance during the summer for CT wheat.

Correlation analysis was performed to examine the relationship between the soil and environmental factors in relation to bacterial abundance for each sampling site (Table 2). All fields had significant correlations to at least one factor, and the correlations differed from those observed in comparison to VLP abundance. For the native prairie, bacterial abundance had significant positive correlations of moderate strength to TopN, OM, and TN (rho 0.40, rho 0.45, and rho 0.49, respectively). Both croplands had a significant positive correlation between bacterial abundance, soil organic matter, and total nitrogen. At the NT canola, bacterial abundance also had a positive significant relationship with soil temperature, leaf area index, and canopy cover. SWC showed a negative relationship with bacterial abundance in NT canola soil (rho −0.32, *P* = 0.0821) and a significant negative effect in CT wheat soil (rho −0.53, *P* = 0.0082).

**TABLE 2:** Influence of soil, plant, and environmental factors on bacterial abundance within fields based on Spearman correlations^*a*^

Parameter	Native prairie		No-till		Conventional till	
	Rho	*P*	Rho	*P*	Rho	*P*
Topsoil nitrate	**0.40**	**0.0392**	0.06	0.3930	0.13	0.2975
Organic matter	**0.45**	**0.0235**	**0.53**	**0.0079**	**0.71**	**0.0002**
Total N	**0.49**	**0.0143**	**0.50**	**0.0125**	**0.69**	**0.0003**
NH_4_	0.16	0.2448	0.04	0.4399	−0.05	0.4177
SWC	−0.07	0.3908	*−0.32*	*0.0821*	**−0.53**	**0.0082**
Avg rain	−0.01	0.5172	−0.31	0.1775	−0.32	0.1701
Min temp	0.24	0.1550	−0.03	0.5501	−0.04	0.5600
Avg temp	0.17	0.2310	−0.11	0.6775	−0.13	0.7044
Max temp	0.19	0.2118	−0.13	0.7131	−0.17	0.7651
Avg soil temp	0.21	0.1900	**0.68**	**0.0469**	0.21	0.2322
Plant biomass	–	–	−0.21	0.3233	0.07	0.5605
LAI	–	–	**0.71**	**0.0454**	0.29	0.7327
Canopy cover	–	–	**0.66**	**0.0481**	0.37	0.2342
Canopy ht	–	–	0.10	0.5636	−0.49	0.1644

a Correlation coefficients with *P* < 0.05 are indicated in boldface; coefficients with 0.1 > *P* > 0.05 are indicated in italics. Dashes (–) represent missing data. Units and abbreviations: topsoil nitrate (lkg/ha), organic matter (%), total nitrogen (%), NH_4_ (lkg/ha), gravimetric soil water content (%), daily average soil temperature at 6 cm depth, leaf area index (LAI), canopy height (cm), canopy cover (%), and dry plant biomass (kg/m2).

The SEM revealed similar results as observed from Spearman correlations (Fig. 3). Several factors appeared to have an influence on bacterial and VLP abundance. Land use had direct significant impact (*P* < 0.001) on bacterial abundance that was not observed for VLP abundance. Land use also had indirect impacts on bacterial abundance by significantly directly impacting NH_4_, TopN, and OM, which further influenced bacterial abundance. In addition, average air temperature had a significant positive interaction with bacterial abundance. While bacterial abundance had a positive impact on VLP abundance, VLP abundance had a significant direct negative impact on bacterial abundance (*P* = 0.001).

### Differences of DNA viral communities between tallgrass prairie and tilled wheat field.

**FIG 4:**
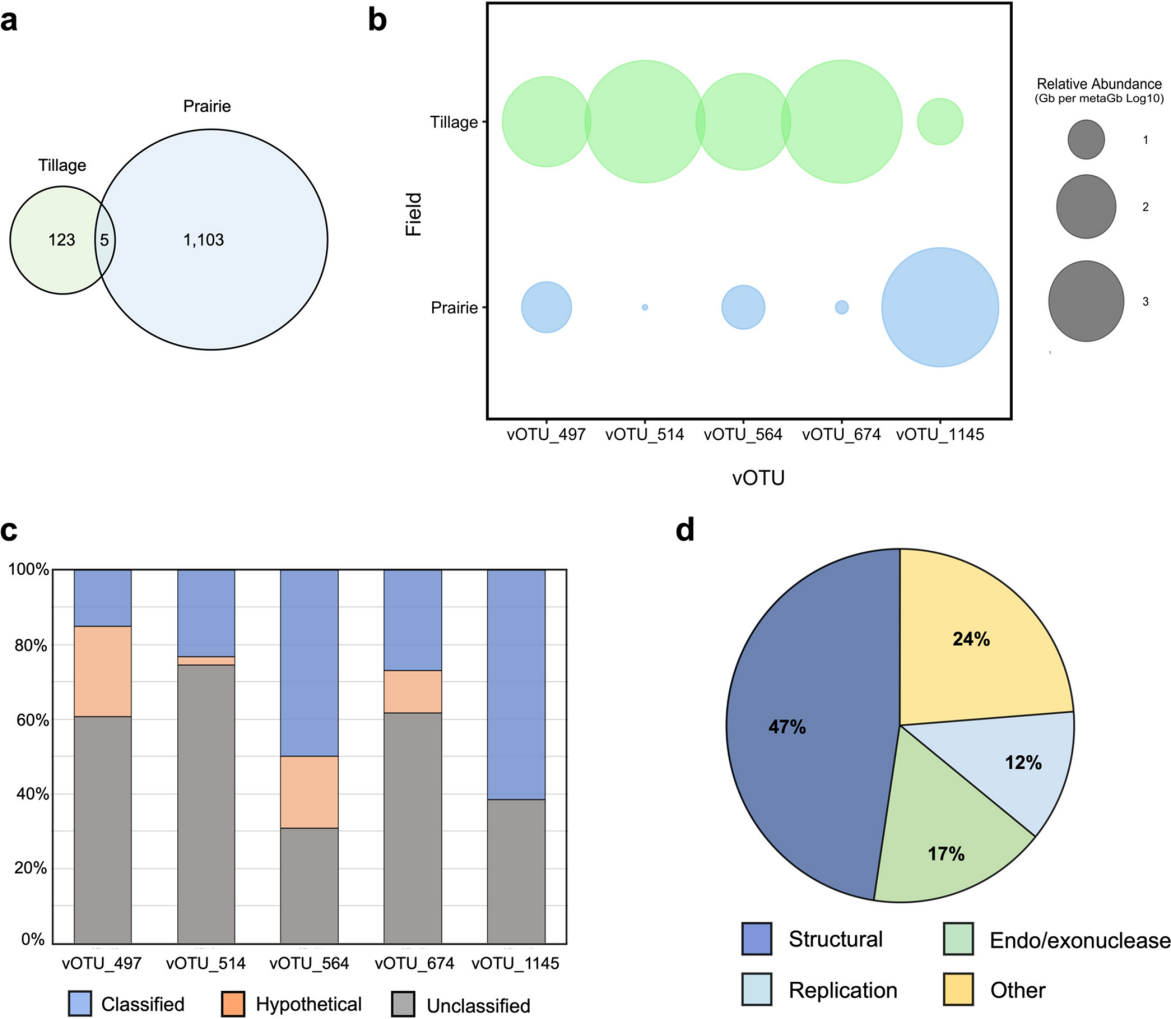
Overlap of viral community structure in soils from different intensities of land management. vOTUs were only considered to be present in an assembly if the vOTU had at least 75% sequence coverage. (a) Depiction of vOTUs in each virome and the number of shared vOTUs between the native and tillage viromes. (b) Bubble plot of the relative abundance of the shared vOTUs in the two viromes. vOTU abundance was normalized by the vOTU length and size of the individual assemblies then standardized by the minimal size of metagenomes (bp) across all samples. (c) Bar graph of portion of predicted genes identified in shared vOTUs. Unclassified genes had no high-quality matches in currently database. (d) Main groups of genes represented from shared vOTUs based on currently available virus protein sequences.

The tallgrass prairie and CT wheat field soils were chosen for metagenomic sequence analysis as they had the greatest differences in VLP abundance, bacterial abundance, and differed the most as far as management input. DNA viral genomes were extracted from purified filtrate enriched with virus particles and sequenced using Illumina technology. A large amount of soil per sample was used for virus extractions and DNA concentrated to avoid amplification methods that might bias sequencing results (38). Metagenome assemblies of viral reads showed observable differences between two sites. The prairie soil virome consisted of 657,863 contigs and the CT wheat field soil virome included 274,051 contigs (Table 3). VirSorter predicted 375 contigs from the CT wheat assembly and 6,856 contigs from the prairie assembly to be possible viruses (≥1 kb). In total, 1,272 viral contigs were over 10 kb, which were used to determine viral operational taxonomic units (vOTUs). For the two data sets, only a little over 3% of the sequences formed clusters with more than one sequence, resulting in 1,231 vOTUs based on 95% average nucleotide identity (ANI) and 80% alignment fraction relative to the shorter sequence. Although the prairie assembly was three times larger than the CT wheat assembly, it had roughly 10-fold more vOTUs identified in the soil virome (Fig. 4a). The majority of the vOTUs were unique to land use type with only five vOTUs shared across assemblies. The relative abundance of the shared vOTUs also differed between the land use types (Fig. 4b). When one of the shared vOTUs was abundant in the CT wheat virome the abundance was reduced in the prairie. The opposite was true as well with vOTUs abundant in the prairie virome being rare in the CT wheat virome. While the richness and Shannon’s diversity index were greater in the prairie, the evenness of the community based on Pielou’s evenness index was relatively similar in the prairie and CT wheat field: 0.925 and 0.908, respectively.

**TABLE 3:** Summary of soil viral metagenomes^*a*^

Metagenome	Total no. of contigs	Total bp	Max contig length (bp)	*N*_50_	VirSorter (≥10 kb)	Total no. of vOTUs
Tallgrass prairie	657,863	831,434,430	227,057	1,450	1,145	1,231^*b*^
Tillage wheat (CT)	274,051	260,104,506	350,802	905	127	

a Virome assembly data provided only includes contigs at least 500 bp in size. VirSorter results represent contigs of **≥**10 kb that were identified as possible viruses from categories 1, 2, 4, and 5. Size-selected sequences were then used to cluster vOTUs using a 95% average nucleotide identity and an 80% alignment fraction.

b Combined total for both tallgrass prairie and tillage wheat.

**FIG 5:**
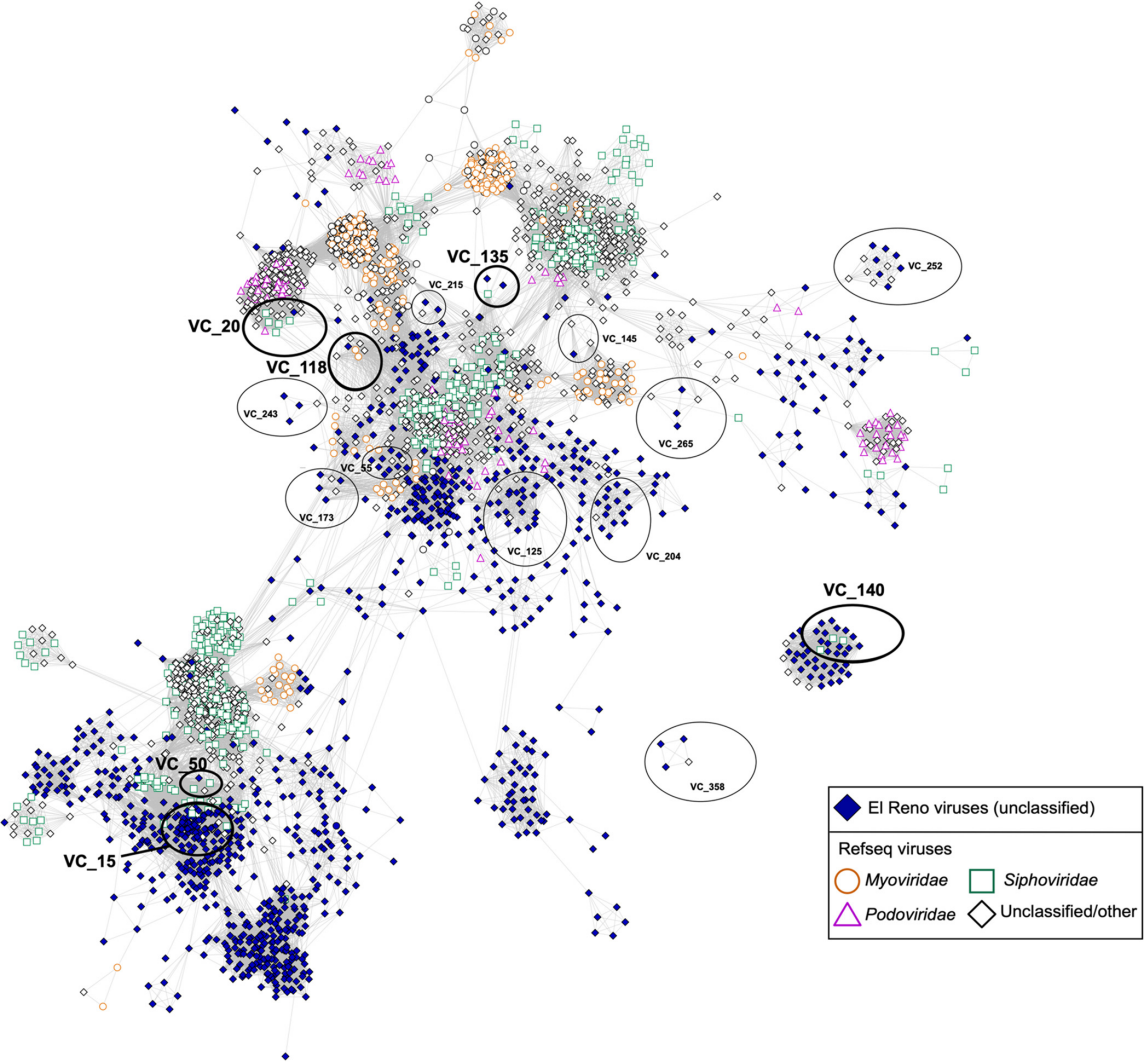
Gene sharing network of El Reno vOTUs clustered with RefSeq viral sequences. Solid colors indicate sequences from El Reno virus data set. Nodes are depicted as shapes of various colors that correspond to virus families within the RefSeq database. El Reno viruses in viral clusters that clearly grouped closely in the network on circled and labeled. Clusters in bolded circles represent those with El Reno viruses that could be taxonomically identified with the family-level taxonomy depicted. Viral clusters that contained only El Reno vOTUs and that did not interact with the main network are not pictured.

vOTUs were grouped into viral clusters (VCs) that were used to predict taxonomy of the viral sequences collected from soils in El Reno, OK. Together, the data will be referred to as the El Reno viruses or vOTUs based on the soil collection location. VCs roughly represent genus-level taxonomy of sequences grouped with a similarity score of at least 1 as previously described (39). Relationships of VCs, including the El Reno vOTUs in comparison to sequences in the RefSeq viral database, are presented in a gene-sharing network (Fig. 5). Of the VCs formed, 34% contained vOTUs from the tillage and prairie soil. The majority of the clusters containing El Reno viruses did not cluster with known viruses in the database and instead formed VCs with sequences in their own data set. Five of the VCs consisted of known viruses from the RefSeq database and El Reno vOTUs. VC_15 consisted of three subclusters, one of which contained all El Reno viruses, and all subclusters grouped closely in the network meaning taxonomically the vOTUs most likely are the same at the family level but not genus-level. The 66 vOTUs in VC_15 were identified as belonging to the family *Siphoviridae*. One vOTU each belonged to VC_20 and VC_50 belonging to the genera *Xp10virus* and *Ydn12virus*, both in the *Siphoviridae* family, respectively. VC_135 belonged to *Ssp2virus* containing two vOTUs and VC_140 belonged to *Pepy6virus* containing 15 vOTUs. Only one vOTU within VC_118 was identified in the *Myoviridae* family belonging to the genus *Msw3virus*. Another 14 VCs containing 76 vOTUs consisted of El Reno viruses that clustered with unclassified viruses in the RefSeq database. The rest of the vOTUs either clustered with other samples in the El Reno data set or were identified as singletons (no significant shared similarity to other protein sequences) or outliers.

Viral OTUs shared between the tallgrass prairie and CT wheat field were compared to known viral sequences to try to determine gene function. Protein coding genes predicted per contig ranged from 13 to 43, with 28 genes predicted on an average. The majority of the predicted genes for the shared vOTUs were not able to be classified or associated with proteins that had no functional identification (Fig. 4c). Of the 141 predicted proteins, 42 were identified and 17 were classified as hypothetical proteins. When looking at the classified viruses, the majority were identified as structural proteins including capsid, tail, and baseplate proteins (Fig. 4d). Proteins related to replication mainly consisted of DNA polymerase and helicase, and endo- and exonucleases made up 17% of the predicted genes. Proteins classified as other did not have enough genes predicted to form clear groups, but several notable proteins include DNA methyltransferase, phage integrase, and hydrolase.

## DISCUSSION

### Virus abundance.

In this study, we investigated VLP abundance over a 1-year period in soils from different land use and management intensities. VLP abundance ranged from 10^8^ to 10^9^ (g^−1 ^dry soil) during the sampling period being consistent with previous research that showed abundance ranging from 10^7^ to 10^9^ (g^−1 ^dry soil) from soils of differing land uses and times of the year (22–25, 40, 41). Specifically in agricultural soils, VLP abundance has been observed between 1.0 × 10^8^ to 1.1 × 10^9^ g^−1 ^dry soil (23–25, 42), which is comparable to the average VLP abundance observed in the croplands in this study. Based on the average VLP abundance for sampling sites, abundance decreased with increasing amounts of land management. The tallgrass prairie had the highest VLP abundance and CT winter wheat soil had the lowest VLP abundance except during one collection month when abundance spiked above that of the NT canola soil (Fig. 2a). According to management data, this is potentially reflective of soil tillage a few weeks prior to the collection that took place during the fallow period. Tillage is a physical disturbance to soil systems that increases soil erosion, loss of soil organic carbon, and loss of aggregate stability (43). However, tillage has also been shown to accelerate microbial activity (44), and increased virus abundance is often thought to occur when there are increased bacterial host activity and abundance in the system (45, 46). This idea was supported by the SEM results of this study where bacterial abundance had a direct positive influence on VLP abundance, and bacterial abundance was directly affected by land use. While increased bacterial host activity and abundance could be responsible for increased VLP abundance in soil, it is not possible to distinguish whether the increased VLP abundance that was observed during certain times of the year was a result of active virus production or virus survival related to physical and/or biological factors (24) in specific soil systems. However, in general, viruses are often impacted by similar factors as their hosts, which affects virus-host interactions (46).

The structural model based on VLP abundance from all study sites showed a clear relationship with nitrate and soil water content both of which were directly influenced by land use. Soil water content has been previously detected to influence virus abundance in other soil systems (23, 40). The soil water content was higher in the prairie and NT canola than in CT wheat, both of which had greater VLP abundance. No-till management can reduce water evaporation from soil and increase water infiltration and soil water content due to plant residues on the soil surface. Survival and inactivation of viruses in soils is often strongly related to wetting and drying of soils (46). Work examining virus survival has shown wetter soils result in virus persistence (47, 48). The CT wheat field had the lowest soil moisture throughout the year due to more water loss through evaporation from soil pores that are exposed directly to radiation. These dry conditions could further contribute to reduced counts in CT soils (46). It is speculated that viruses are likely to passively distribute with water, and due to their size are expected to be present in micro- and nanoscale soil pores (49). The increased presence of organic matter can improve soil water holding capacity and overall soil structure (50). The organic matter content of the soil is also considered to be linked to increased VLP abundance. While no direct interaction was found between OM and VLP abundance in this study, organic matter was directly influenced by land use and impacted bacterial host abundance (Fig. 3). The greater levels of organic matter in prairie soil may further explain the increased virus survival, partially due to the input of fertilization from grazing cattle throughout the year. The CT wheat field also had grazing cattle for the portion of the year, but there was no observable effect on virus abundance. Other more intense management practices likely had a greater overall impact on the virus community. Previous studies have reported agricultural soils having lower VLP abundance than nutrient-rich forest and pasture soils, both of which were associated with organic matter and water content (24, 25, 42). Land use and management practices also greatly affect soil temperature. Although little is known about the persistence of autochthonous viruses in soils, laboratory incubation experiments introducing nonnative viruses to soil demonstrated temperature was a key factor controlling virus survival in soil with survival often being favored at cooler temperatures (47, 48, 51). Tallgrass prairies accumulate an enormous amount of biomass leading to thick groundcover in contrast to croplands where above ground biomass is removed yearly, exposing the soil. More groundcover is present in an NT system where residues are left on the soil surface compared to CT management where residues are incorporated into the soil. Overall, tillage management leaves the soil more vulnerable to the elements for a larger portion of the sampling year. Based on correlation analysis, larger plant-related measurements reflective of land cover have a positive relationship with virus abundance. The presence of crops and crop residue may have played a role in virus survival by relieving stress especially in the form of high soil temperatures throughout the year. Although this is just one possibility, increased virus presence could also be based on greater bacterial host abundance (34, 52) or virus lifestyle choice based on host nutrient availability (53, 54). While already known to influence microbial communities (55), nitrogen levels in the soil also impacted the virus populations. Nitrate was an important factor linked to overall virus abundance (Fig. 3). The response to different nitrogen sources based on Spearman’s correlations in relation to viral abundance in the two croplands might be reflective of the differences in the viral and host community composition and their function in the soil system. This is supported by our model that confirms the important influence on nitrate in the soil by NH_4_, air temperature, and management practices. Together, this study and prior studies of viral diversity demonstrate that local environmental conditions have a strong effect on the viral community (25, 42, 56, 57). Therefore, it is highly likely that viral abundance and community structure are in part shaped by the biotic and abiotic factors influenced by land use and land management practices.

The VLP abundance data show temporal variation over the 1-year collection period that was often observed during specific months instead of across seasons. A 12-month study by Narr et al. also detected seasonal differences and changes in abundance over time in the majority of their sampling sites (25). When looking at growing seasons alone, a similar study found viral abundance to be roughly constant from May to July and September to November in a range of agricultural treatments (42). Overall, this resembles what is observed during the growing seasons of both croplands in this study (Fig. 2a), but the largest temporal difference we observed in VLP abundance occurred in March when the weather begins to warm up and crops resume actively growing. Long-term studies examining VLP abundance in soil are very limited; therefore, more studies are needed to support these initial findings. However, variation in seasonal VLP abundance was recorded early on in viral studies in seawater (20). Many marine viral studies have observed changes in abundance by an order of magnitude between the winter and the summer months (27, 58). While temporal differences were observed in our study, the scale of the change in soils appears to be much smaller than that of marine systems (25, 42). The amount of variation in VLP abundance throughout the year differed for each field. The temporal responses were larger in prairie soil, but all fields had lower abundance in winter months and higher abundance during March. Although VLP abundance had similar temporal dynamics, the differences observed in the magnitude of the variation are likely due to increased management activity that continually disturbs the system, including the microbial hosts needed for virus production.

### Bacterial host abundance.

Bacteria are speculated to be the most common hosts for viruses in environmental samples, explaining why bacterial abundance is often examined in combination with viral abundance. In this study, estimates of bacterial abundance ranged from 10^6^ to 10^9^ bacterial cells g^−1^ soil. Although approaches differed, this is consistent with other investigations that determined bacterial abundance to be ≥10^6^ g^−1^ soil (24, 40, 42). As seen with VLP abundance, there were significant differences between bacterial abundance in the cropland soils in comparison to the tallgrass prairie soils. Changes in abundance followed the same structure observed in the viral communities such that bacterial abundance decreased with increasing land management. Considering the idea that most viruses present in soils are bacteriophages, it is not unexpected that the observed population abundance for viruses and bacteria responded in a similar manner to land use and land management practices. Numerous studies in marine systems have looked at bacterial abundance and its relationship to viral abundance. In these systems, VLP abundance is highest in coastal environments and lowest in deep-sea waters in general (18). These variations in abundance are often correlated with microbial production and the productivity of the system (24, 46). Soil studies have demonstrated similar relationships where organic-rich soils with higher moisture have greater prokaryotic cells present than that of dry low organic content soils; the latter of which generally results in a much greater presence of virus than prokaryotic hosts (3, 23, 24, 59). As seen here using SEM, the positive direct influence of bacterial abundance on VLP abundance suggests increased productivity of bacteria is advantageous for viruses, while the negative direct effect of VLP abundance on bacterial abundance implies increase in virus abundance is unfavorable for the host population. Most current soil studies do not look specifically at bacterial cell counts, but a large number have shown that soil microbial communities are considerably affected by changes in land use and land management (55, 60–62). Such changes in land use and management also have a significant effect on soil and environmental factors (46, 63, 64), all of which could contribute to the differences in bacterial abundance observed between the different sites.

Microbial activity and biomass have been shown to respond to multiple influences, including organic matter, soil management, and other abiotic factors (15, 24, 25, 65). Our data also indicate that many factors, including ground cover, soil nutrients, soil water content, and temperature, all of which are influenced by land use and land management, have significant interactions with soil bacterial abundance. The CT cropland had the most significant correlation between soil water content and bacterial abundance. The water content fluctuated more in the croplands than the prairie soil over the sampling period. Notably, bacterial abundance was negatively related to SWC, while VLP abundance had a positive relation with SWC, which may reflect virus production in the soils. Most often higher moisture in soil supports an increase in bacterial activity and abundance (66, 67), but an increase in the activity of a typically starved host infected with a virus can lead to induced virus production and host lysis (46, 68). It is also possible that increased water in the soil dilutes or mobilizes the microbial hosts especially in the loose soil of the tillage site, although this would be expected to be accompanied by an even greater runoff of viruses (69).

Soil microbes play an important role in nutrient cycling, decomposing organic matter, carbon mineralization, and plant nutrient availability (70, 71). These differences in functional activities of microbes are impacted by land cover which differs substantially between land uses. In addition, land cover has been found to regulate microbial structure by affecting soil conditions such as organic matter (72, 73). Bacterial abundance was strongly influenced by organic matter and total nitrogen at all sites based on Spearman’s correlations, both of which strongly decreased with land cover and increased with management intensity. Land cover is also known to be a controlling factor of soil temperatures that is overall influenced by air temperature. Bacteria and virus survival in soil is often temperature dependent, and optimal temperatures can differ between hosts and their associated phages (46). In the cropland sites, the directions of the relationship with temperature overall differed for bacterial and viruses. The changes in soil bacterial abundance may result from prophage induction triggered by increased temperature resulting in host cell lysis. Virus production can be induced by an environmental signal such as host DNA damage, resulting in the lytic function of lysogenic viruses and the production of progeny (74). For example, DNA damage can induce an SOS repair mechanism initiating the lytic pathway of virus replication in lysogens (75). Alternatively, it could be caused by selective mortality of different microbial groups which have been recently shown to be triggered by bacterial quorum sensing signals inducing a lysogenic to lytic switch in samples collected from agricultural soils (76); any of these could result in the different response in abundance to changes in soil and air temperature by the bacterial and virus populations. Nitrate and ammonium both were key in determining bacterial abundance based on SEM and Spearman’s correlations. These two forms of nitrogen, especially at elevated levels from fertilizer input, impact soil processes and shape microbial community structure (77). While the changes observed depend on the specific land use and management practices, sampling site overall appears to have the largest impact on soil factors which affects the below ground community dynamics.

There were observable seasonal shifts in bacterial abundance at all sampling locations over the 1-year study period. The lowest bacterial abundance was recorded in August and the following winter months for all fields similar to the temporal lows in VLP abundance. Increases in bacterial abundance were observed in fall and spring extending into early summer. While all the fields had similar shifts in abundance throughout the year, the magnitude of the changes varied greatly. Comparable results were detected in agricultural soils in Michigan where bacterial abundance stayed relatively stable and quickly returned to these stable levels when fluctuations in abundance occurred, but this study did not account for differences in bacterial abundance during winter and summer months (42). The same marine studies that observed seasonal changes in VLP abundance also observed similar changes in bacterial abundance, but of smaller magnitude than that seen for viruses (27, 58). Although the results are from contrasting systems, the same general variations appear to be present in the recent studies of soil systems. Further studies of combined viral and bacterial abundance are needed to determine the seasonal effects on virus and host interaction in terrestrial systems.

### Viral community.

The scarcity of studies examining viral communities especially in terrestrial environments is usually attributed to the absence of a genetic marker sequence, such as those used in identifying bacterial communities (78). Certain viral taxonomic groups share conserved genes which allow them to be used as targets to study specific viral groups (56), but a less targeted approach needs to be used to look at the whole community. Fingerprinting methods have allowed for fast analysis and higher sample throughput for screening viral communities but lack information on viral abundance and identity (25). For these reasons, most examinations of viral community structure rely on metagenomic approaches. Studies have recently started to focus on optimizing protocols for viral metagenomic analysis from terrestrial environments in order to create a standardized method for viral communities to be compared across environments (39, 79, 80). However, it should still be taken into consideration that soil, environmental, and viral factors are known to affect the adsorption of viruses to soils (46), and virus extractability from soil can be further impacted by the extraction method (24, 25, 40). Viral metagenomics provides more than just sequence data by offering insight into biogeographical distributions, community structure, and ecological dynamics (78).

In order to determine the possible impacts of viruses on soil microbial communities, it is critical to study autochthonous viruses using cultivation-independent approaches to assess community composition. Current bioinformatic tools were used to characterize viruses in soils under different levels of management intensity. These recent tools have provided a way to use assembled virus fragments that have not been previously cultivated or identified in phylogenetic and diversity studies (37). In El Reno soils, the majority of identified viral sequences did not cluster together, suggesting the majority of the sequences represented unique virus species or vOTUs. The large assembly and greater number of viral species in the native tallgrass prairie is also consistent with the observations of greater VLP abundance in the prairie soil. The term viral operational taxonomic units (vOTUs) has been proposed at the formal way of classifying species-rank virus groups in order to streamline the area of viral ecology and prevent confusion between various terms used across studies (37). There was also very few shared vOTUs between the two land use types as has been previously observed in other habitat gradients (36), supporting the idea that viral communities are influenced by the environment in which they are found.

Clustering methods of comparing new viral data sets to known viruses in available databases provides a way to examine relationships between identified and unknown viruses while assigning taxonomic classification to uncultivated virus genomes (UViGs) (37). UViGs represent the majority of virus sequences in available databases due to the use of metagenomic and metatranscriptomic studies (37, 81–83). By clustering the vOTUs from El Reno with publicly available viruses, we were able to identify 86 of vOTUs from assembled viromes with another 76 vOTUs grouping with unclassified viruses in the RefSeq database. The majority of vOTUs from CT wheat and native prairie soils had no genetic similarity to viruses in the current databases. Similar results have been obtained in other studies where only 8.5 to 24.3% of viral sequences were identified in Chinese agricultural soils (84), 9.8% in polar freshwater (85), and 15% thawing permafrost harbors (36). In combination, these studies reveal the limitations of examining viral communities showing most viromes consist of predominately unidentifiable sequences. This was further exemplified when examining proteins in a subset of the El Reno vOTUs, where less than half of the predicted genes were identified based on currently available sequences. Each field’s taxonomic profile differed by the presence of specific bacteriophage families and the relative abundance of taxonomically identified viruses. *Siphoviridae* was the dominantly identified group in both viral communities with *Podoviridae* not being identified in either virome, but due to the lack of identified viruses in the El Reno viromes it is hard to determine which specific viruses are abundant in the community. Although, it does appear that each virome is distinct to the collection site with there being little overlap in the viral community structure, which could be partially due to the technical issues associated under sampling and reproducibility (86–88). When examining the shared vOTUs, unique function was not observed most likely due to the lack of predicted gene identification. One shared vOTU highly present in the tilled soil contained methyltransferase genes which are known to be a powerful gene regulator in bacteria and have been proposed as a life cycle regulator in phages (89). Switching life cycles in soils subject to frequent disturbances could be an important and distinctive function in frequently disturbed soils such as intensely managed croplands. Comparably, earlier soil viral metagenomic data have revealed that viral assemblages are locally unique, and medium type is most likely the driving force behind observed differences when comparing viral communities (56, 84). More specifically, the texture and physiochemical factors may influence the community more than distance between sites (84), supporting the idea that viral abundance and community structure are influenced by various soil and environmental factors which are known to be affected by land use and land management practices. Although there were clear observable differences in VLP abundance, vOTU abundance, and community structure in the two fields, further work is required to determine whether similar environmental factors and seasonal differences are influencing the community structure over time as was observed for virus abundance.

### Conclusions.

In each land use system, there were clear temporal differences in viral and bacterial abundance over the 1-year sampling period. The abundance of viruses and potential hosts both decreased with increasing amounts of management input with the prairie site continually having greater abundance than the croplands. There were also observable seasonal differences in abundance with similar trends for virus and bacterial populations. Various soil and environmental factors influenced viral and host abundance which was often reflective of management activities in each system. When examining DNA viral communities in the prairie and tilled wheat field, there were clear differences in community structure and vOTU relative abundance with the native tallgrass prairie containing more unique viral species. There was also minimal intersection of the viral community structure between land use types. This study suggests that the different levels of land management impacted the soil properties and environmental effects on the below ground communities especially abundance. Overall, our results implicate land use and land management as driving factors of shaping the physicochemical properties in agricultural soils which influence not only the abundance of virus and host communities but the structure of the soil viral communities. Global or large-scale studies are needed to identify whether such interactions between management, environmental factors, and viruses are a general rule across all agricultural systems.

## MATERIALS AND METHODS

### Sample sites.

Soil samples were collected at the U.S. Department of Agriculture, Agricultural Research Service, Grazing Research Laboratory in El Reno, OK (35°34.1′N, 98°03.6′W; 414 m above sea level), from August 2016 to October 2017. Samples were taken approximately every 4 weeks from a native tallgrass prairie (35°32.9′N, 98°02.2′W; 64 ha), conventional till (CT) winter wheat (35°34.1′N, 98°03.3′W; 27.5 ha), and no-till (NT) winter canola (35°34.07N, 98°03.5W; 20.5 ha). The croplands and prairie sites were ∼2.7 km apart. The native tallgrass prairie was native, mixed species grassland managed by cattle grazing several months out of the year and spring burns on a 4-year rotation with the most recent burn occurring in 2014. The soil was classified as Norge loamy prairie (fine, mixed, thermic Udertic Paleustalf) with a high-water holding capacity and a depth of >1 m (90). Winter wheat fields represent a cool season crop that dominates in central Oklahoma in areas where tallgrass prairies have been converted to croplands. The soil type at the croplands was characterized as Bethany silt loam (fine, mixed, superactive, thermic Pachic Paleustolls) (17). In Oklahoma, winter wheat fields are managed for multiple purposes (grain production and cattle grazing). The CT wheat field was managed for grain production (grain-only) during the 2015-2016 growing season and graze-out wheat (no grain production; cattle grazing from November through May) during the 2016-2017 growing season. Each year the seedbed was prepared for planting using a chisel plow treatment to a depth of 31 cm, which resulted in complete disturbance of soil and residue mixing (17). The NT cropland field was grain-only wheat during the 2015-2016 growing season and on canola rotation during the 2016-2017 growing season. No-tillage treatment was initiated in 2015 only (just a year prior to this experiment). Detailed management data have been previously published (91). For each soil sampling time point, eight cores roughly 20 m apart were taken in a random walking pattern throughout each field at a depth of 0 to 15 cm using a 2.5-cm-diameter soil probe. Soil cores were pooled and homogenized to deal with soil heterogenicity and sieved to 2 mm to remove debris prior to analysis. Soils were kept on ice and directly transports to the lab where they were kept at 4°C for virus extraction, while soils for bacterial and chemical analysis were stored at −80°C. Samples for virus extraction were stored for a maximum of 48 h before processing. Not all soils were used in every experiment.

### Environmental, soil, and plant data.

Weather data were gathered from the Oklahoma Mesonet station (http://www.mesonet.org/index.php/weather/local/elre) in El Reno (ELRE), OK. The Mesonet tower is located on the native tallgrass prairie used in this study at 35°32.9′N and 98°02.2′W. Data used from Mesonet measurements included average rainfall, maximum air temperate, average air temperature, and minimum air temperature. Similar weather data for croplands were collected from eddy covariance stations located in those fields. Soil chemical analysis was performed at the Oklahoma State University Soil, Water, and Forage Analytical Laboratory (http://soiltesting.okstate.edu/). Tests included topsoil nitrate, organic matter, total nitrogen, and ammonium. Gravimetric water content was determined by oven drying for ≥24 h at 65°C or until the weight no longer changed (17). Leaf area index (LAI) was measured nondestructively using an LAI-2200C plant canopy analyzer (LI-COR Inc., Lincoln, NE), and the percent canopy cover (Canopy%) was determined using the Canopeo app. The aboveground biomass was collected destructively from five randomly located 0.5 × 0.5 m^2^ quadrats within each field at 2-week intervals during the active growing season. Dry biomass weights were recorded after drying samples in forced-air oven at 70°C for a minimum of 48 h (91).

### Bacterial extraction and qPCR.

Bacterial genomic DNA was extracted with a Quick-DNA fecal/soil microbe miniprep kit (Zymo Research, Irvine, CA) according to the manufacturer’s protocol with the exception of eluting DNA with sterile water. For each pooled soil sample, four subsamples were used for extractions. DNA was quantified with a Quibit dsDNA BR assay kit (Thermo Fisher Scientific, Waltham, MA) as described by the manufacturer’s instructions. DNA dilutions of 2 ng/μl were prepared to use for downstream analysis. qPCR was performed to estimate bacterial abundance based on the copy number of 16S rRNA genes using an Applied Biosystems 7300 real-time PCR system (Thermo Fischer Scientific). All four replicates were run for each sampling time point and collection site. PCR was performed in a total volume of 30 μl that contained 15 μl of Power SYBR Green Master (Thermo Fisher Scientific), 2 μl of DNA template, and 100 nM concentrations of primers 27F and 519R (92, 93). The qPCR thermocycling steps included 95°C for 10 min, followed by 40 cycles of denaturation at 95°C for 45 s, annealing at 55°C for 45 s, and extension at 72°C for 1 min. The *C_T_* (threshold cycle) and 10-log-fold standard curves were used to estimate bacterial abundance in soils by converting *C_T_* values into estimates of bacterial cells present in 1 g of soil in each technical replicate. The amount of template DNA used for qPCR and the amount of soil used for each DNA extraction were accounted for in abundance estimates. Estimates were then converted to cells per gram of dry weight. Negative controls had no detectable amplification.

### Virus-like particle extraction.

Viruses were extracted from soil samples using an adaptation of the method by Williamson et al. (23, 24). In short, 5.0 g of fresh soil was weighed into acid-cleaned 50-ml glass test tubes containing 15 ml of sterilized potassium citrate buffer (10 g of potassium citrate, 1.44 g of Na_2_PO_4_, and 0.25 g of KHPO_4_ [pH 7.0] per liter) stored at 4°C. Viruses were mechanically separated from soil samples through sonication. Each tube was sonicated using a Branson 5510 ultrasonic for a total of 10 min with vortexing at high intensity for 20 s every 2 min. Samples were centrifuged at 8,000 × *g* for 25 min at 4°C to sediment large soil particles. Supernatants were filtered through a 0.2-μm syringe filter (GE Healthcare Life Sciences, Marlborough, MA) to remove bacteria and other large particles, and the filtrate was collected into sterile 15-ml polypropylene tubes and stored at 4°C. Three subsamples were used for VLP extraction from each composite field sample.

### Epifluorescence microscopy quantification of VLPs.

For VLP enumeration, 1 ml of viral extract that had been diluted at a 1:4 ratio with sterile deionized water was vacuum filtered through a 0.02-μm Anodisc filter (25 mm diameter, Whatman International, Ltd., Maidstone, England). A 0.45-μm filter (Pall Life Sciences, Port Washington, NY) was used for support. Anodisc filters were stained with 500 μl of 2.5 × SYBR gold (Invitrogen/Thermo Fisher Scientific, Waltham, MA) in the dark for 15 min. Excess SYBR gold was vacuumed through, and filters were washed with 1 ml of sterilized TE buffer. Filters were then mounted on glass slides using 30 μl of antifade solution on the coverslip (23) and analyzed by epifluorescence microscopy using an Olympus BX61 motorized system microscope with an attached DP71 digital camera (Olympus Corp., Center Valley, PA). Three slides in total were made for each field and time point, one from each replicate extraction. The number of VLPs were counted manually in 10 fields per slide at ×1,000 magnification. The average VLP counts were calculated from the grand mean of the replicate filters per gram of dry soil (23, 24).

### Virus dsDNA extraction and sequencing.

Large soil samples (∼500 g) were collected from the native tallgrass prairie and conventional tillage winter wheat site in October 2017 for viral DNA extraction. Using 200 g of fresh soil per field, soil samples were treated as described above to extract VLPs for the purpose of DNA extraction. VLPs were then pelleted using an Optima LE-80K Ultracentrifuge (Beckman Coulter, Brea, CA) and a SW 28 Ti swinging bucket rotor at 50,000 rcf for 2 h at 4°C in thin-wall, Ultra-Clear, 38.5-ml centrifuge tubes (Beckman Coulter). For each soil sample, six tubes containing 0.2-μm-filtered supernatant were centrifuged. Pellets were resuspended and combined in 200 μl of potassium citrate buffer. Samples were treated with DNase (100 U/ml) to remove any free contaminant DNA before lysing the virus particles (94). DNase reactions were stopped by incubating samples at 65°C for 10 min in the presence of 0.5 M EDTA. Viruses were lysed using 1 volume formamide, 0.1 volume 2 M Tris-Cl, and 0.05 volume 0.5 M EDTA at 37°C for 30 min (95). DNA was then collected by PEG precipitation as described by Sambrook and Russell (96). Pelleted DNA was resuspended in 200 μl of sterile water. dsDNA was extracted by using a Quick-DNA fecal/soil microbe miniprep kit (Zymo Research, Irvine, CA) according to the manufacturer’s instructions with the exception of removing the bead-beating lysis step. DNA was quantified using a Quibit dsDNA BR assay kit (life Technologies/Thermo Fisher Scientific) as described by the manufacturer’s protocol. DNA was sequenced using Illumina HiSeq PE150 technology at the Oklahoma Medical Research Foundation.

### Bioinformatic analyses.

Raw reads for each metagenome were evaluated for quality using FASTQC (https://www.bioinformatics.babraham.ac.uk/projects/fastqc/), and duplicates were removed by using CD-HIT (97). Reads were then quality trimmed and filtered using the NGCS QC Toolkit (98). IDBA_UD (99) was used for metagenome assembly using default parameter and keeping contigs 500 bp or larger. Using CyVerse, assemblies were processed with VirSorter to determine viral sequences using the Virome database (100). Sequences from VirSorter categories 1, 2, 4, and 5 were kept (35, 100). Contigs of ≥10 kb were selected and clustered into vOTUs using the CyVerse app ClusterGenomes (v1.1.3) with the parameters 95% average nucleotide identity and 80% alignment fraction of the smallest contig (82). vOTU relative abundance was estimated by mapping reads using Bowtie2 (101) with multimapping and zero mismatches (39). vOTUs were only considered present in a sample if at least 75% of a contig was covered. To normalize each data set for comparison, the total number of base pairs mapped were divided by the vOTU sequence length and divided by the total number of base pairs in the metagenome (36). Bubble plots of the relative abundance of vOTUs was constructed using ggplot2 in R version 3.6.1 (102). Taxonomic classifications were determined by vContact2 by producing viral clusters (VCs) based on viral predicted proteins with pairs of sequences with a similarity score of >1 being clustered into viral clusters (39, 82, 103). Reference sequences that coclustered with soil viral sequences from the present study were used to predict the taxonomy using the last common ancestor approach and if the taxonomy of reference genomes within a VC differed, majority rule was used (39). The network was then visualized and imaged using Cytoscape v3.8.0 (104). MetaProdigal was used to predict open reading frames (ORFs) for the shared vOTUs. The predicted proteins were then compared the viral RefSeq database using a minimum bitscore of 50 using blastp. Protein searches were also done using NCBI virus (https://www.ncbi.nlm.nih.gov/labs/virus/vssi/#/), which includes virus sequences not available in the RefSeq database. Up to the top 500 query results for each ORF was compiled into a custom database, and blastp was used again to compare the proteins to the custom database. Results from both searches were compared to determine the best match for each gene prediction.

### Statistical analysis.

Principal-component analysis was performed using soil chemistry data for the three collection sites in R version 3.6.1 (102). To test for significant differences of soil chemistry between sites, data were checked for normality than analyzed using the aovp function in the lmPerm R package. Differences were considered significant base on a *P* value of ≤0.05. *t* tests were used to compare plant biometrics data for the fall and spring growing season. Spearman correlations were calculated using the cor.test function to determine the relationship between viral abundance, microbial abundance, soil properties, and other environmental factors. Correlations were done separately for each field due the difference in soil chemistry for each sampling site. The rho value for moderate to very strong correlations range from 0.4 to 1.0, while significant correlations were determined by a *P* value of ≤0.05. Relationships for the abundance, soil chemistry, and air temperature were further examined with structural equation modeling (SEM) using the lavaan package in R. Tallgrass prairie data were treated as the control with CT and NT management were used as treatments.

### Data availability.

Raw metagenomic data for each viral metagenome was deposited in the Sequence Read Archive (SRA) database under BioProject accession number PRJNA669149.
